# Multiple endocrine neoplasia 2A presenting in a family with a history of Hirschprung’s disease

**DOI:** 10.1093/omcr/omab122

**Published:** 2021-12-28

**Authors:** Allison M Johns, Angela H Vuong, Omer A Hassan, Reese W Randle, Matthew A Gorris

**Affiliations:** 1 Division of Endocrinology and Metabolism, Atrium Health Wake Forest Baptist Medical Center, Winston-Salem 27157, USA; 2 Department of Internal Medicine, Atrium Health Wake Forest Baptist Medical Center, Winston-Salem 27157, USA; 3 Department of Pathology, Atrium Health Wake Forest Baptist Medical Center, Winston-Salem 27157, USA; 4 Department of General Surgery, Atrium Health Wake Forest Baptist Medical Center, Winston-Salem 27157, USA

## Abstract

Hirschprung’s disease co-occurs with multiple endocrine neoplasia type 2A infrequently but at a higher rate with certain RET mutations. We present a case of a patient evaluated for an adrenal incidentaloma with a history of familial Hirschprung’s. Our patient was found to have synchronous pheochromocytoma and medullary thyroid carcinoma illustrating the importance of genetic testing in these patients to determine appropriate screening for endocrine tumors.

## INTRODUCTION

Multiple endocrine neoplasia type 2 (MEN2A) is an inherited disorder involving the RET proto-oncogene and is associated with an increased risk of developing a variety of endocrine tumors. Activating RET mutations can be found in up to 5% of sporadic Hirschprung’s cases and up to 50% of familial cases [1, 2]. Hirschprung’s disease (HD) and MEN2A co-occur relatively infrequently but have been found to occur at a higher rate in those with certain RET mutations [2]. The presentation of Hirschprung’s is more often seen in the newborn period [3], whereas thyroid carcinoma and pheochromocytoma of MEN2A typically present later in life, though still earlier than with sporadic cases. We present a case of a patient presenting for an adrenal incidentaloma with a history of HD as an infant.

## CASE REPORT

**Figure 1:**
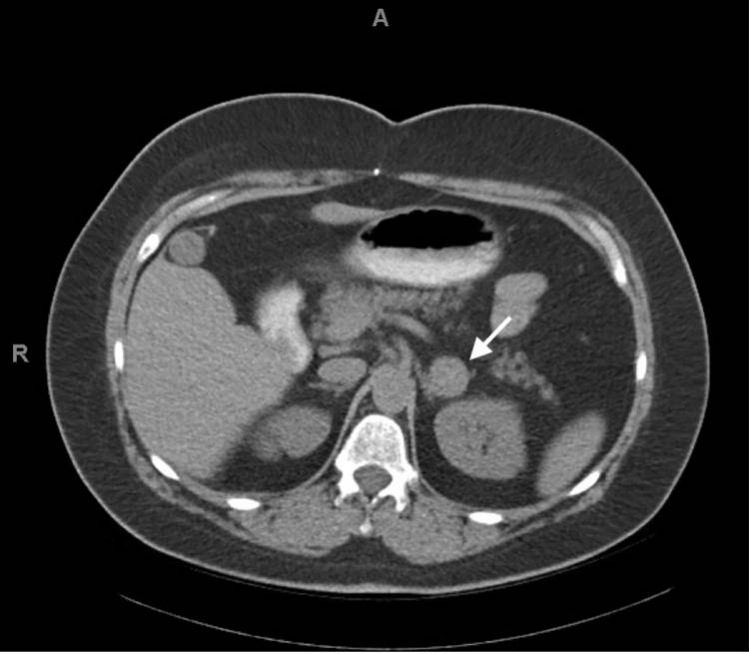
CT abdomen and pelvis without contrast demonstrating high density (40 HU), 2 cm left adrenal incidentaloma.

Our patient is a 45-year-old female who presented for the evaluation of adrenal incidentaloma. Her medical history was significant for type 2 diabetes as well as a personal and familial history of HD, for which she underwent several abdominal operations. Computed tomography (CT) performed to evaluate bulging around her colostomy site incidentally discovered a 2 cm round, uniform mass in the left adrenal gland with a density of 40 Hounsfield units on non-contrast images (Fig. 1). At her presentation to clinic, she reported 1 year of occasional episodes of diaphoresis and palpitations. She was normotensive with a heart rate of 87 at initial visit. Prior to her initial visit, a basic laboratory and adrenal incidentaloma workup had been initiated (Table 1), which was significant for elevated urine metanephrines of 827 mcg/24 hours confounded by excess caffeine use and duloxetine, which she takes chronically for depression. Plasma metanephrines after withdrawal of caffeine and duloxetine were still elevated two times the upper limit of normal (metanephrine 0.96 nmol/l (normal < 0.5 nmol/l), normetanephrine 0.91 nmol/l (normal < 0.9 nmol/l)). Given concern for Hirschprung’s variant of MEN2A, serum calcitonin was obtained and elevated to 120 pg/ml (normal < 7.6 pg/nL).

**Table 1:** Initial laboratory workup

Lab Finding	Value	Reference range
WBC (×10^3^/μl)	10.4	4.0–10.5
RBC (×10^6^/μl)	5.11	3.87–5.11
Hemoglobin (G/dl)	14.8	12.0–15.0
Hematocrit (%)	42.8	36.0–46.0
Platelets (×10^3^/μl)	327	150–400
Absolute neutrophils (×10^3^/μl)	6.4	1.7–7.7
Absolute lymphocytes (×10^3^/μl)	3.2	0.7–4.0
Absolute monocytes (×10^3^/μl)	0.5	0.1–1.0
Absolute eosinophils (×10^3^/μl)	0.2	0.0–0.7
Absolute basophils (×10^3^/μl)	0.1	0.0–0.1
Glucose (mg/dl)	104	70–99
TSH (UIU/ml)	1.79	0.450–5.330
24-hour urine free cortisol (mcg/24 hour)	14	3.5–45
24-hour urine metanephrines (mcg/24 hours)	**827**	30–180
24-hour urine normetanephrines (mcg/24 hours)	**556**	119–451
24-hour urine total metanephrines (mcg/24 hours)	**1383**	156–561
Plasma free normetanephrines (nmol/l)	**0.91**	<0.90
Plasma free metanephrines (nmol/l)	**0.96**	<0.5
Calcitonin (pg/ml)	**120**	<7.6

**Figure 2:**
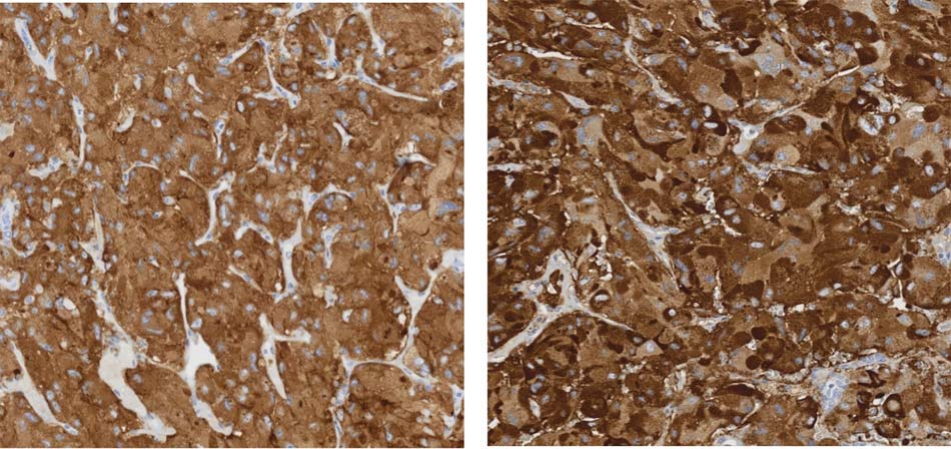
Immunohistochemical staining for synaptophysin (left) and chromogranin (right) shows strong reactivity in cells of pheochromocytoma.

**Figure 3:**
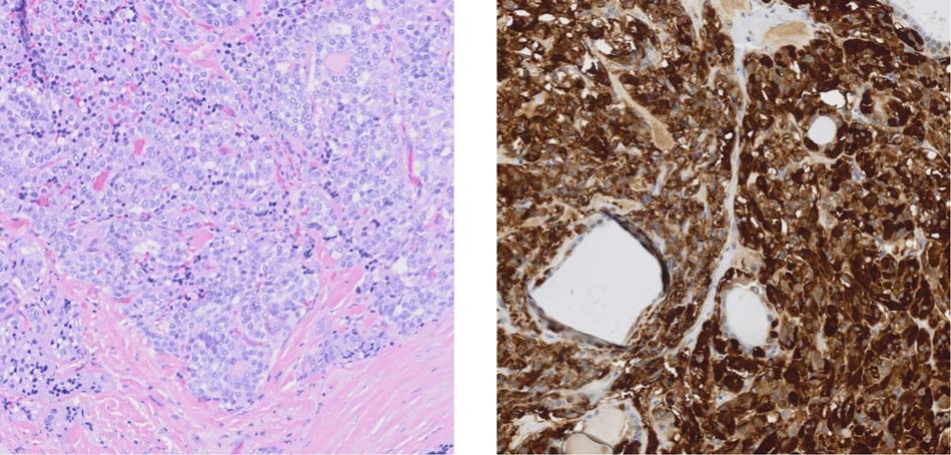
Medullary thyroid carcinoma showing variably sized nests composed of cells exhibiting round to ovoid nuclei with coarsely granular chromatin and moderate eosinophilic cytoplasm. A characteristic background of dense eosinophilic stoma containing amyloid is noted (left). Immunohistochemical staining for calcitonin (right) shows strong reactivity in the cells of medullary thyroid carcinoma.

Thyroid ultrasound demonstrated no distinct thyroid nodules or concerning adenopathy but did find hypoechoic areas posteriorly on both sides of the thyroid. Genetic screening revealed RET c1.1858T>C (p.Cys620Arg). She underwent left retroperitoneoscopic adrenalectomy followed by total thyroidectomy and bilateral central neck dissection. Pathology confirmed both pheochromocytoma (Fig. 2) as well as multifocal medullary thyroid carcinoma with 4 of 12 lymph nodes positive for disease (Fig. 3). The patient’s genetic testing positive for RET mutation, as well as pheochromocytoma, medullary thyroid carcinoma, and history of HD confirmed a diagnosis of MEN2A.

After the diagnosis of MEN2A, the patient was counseled to discuss this with family members and pursue evaluation in our clinic and genetic testing. Our patient’s maternal great grandfather reported a history of Hirschprung’s as well as multiple siblings of her mother who passed away before the age of one due to complications of Hirschprung’s. The patient’s mother was found to have the same mutation as her daughter. She underwent appropriate tumor screening and was diagnosed with synchronous bilateral pheochromocytomas, medullary thyroid cancer, and primary hyperparathyroidism.

The patient recovered after her surgeries with normalization of her metanephrines. However, 12 months after her thyroidectomy, she continues to have an elevated calcitonin of 33 pg/ml (ref range < 7.6 pg/ml), suggesting persistent medullary thyroid cancer that may eventually require additional therapy in the future. Imaging thus far has not yet identified any sites of metastatic disease.

## DISCUSSION

Up to 5% of patients with sporadic Hirschprung’s and 50% of familial cases have MEN2A; however, because these patients do not routinely undergo genetic testing, there is a high likelihood that the diagnosis will be delayed or missed. Establishing the diagnosis of MEN2A allows for regular screening and even prophylactic thyroidectomy due to the high risk of medullary thyroid cancer. A thorough medical history should be taken from any patient being evaluated for thyroid cancer or pheochromocytoma to ensure a history of Hirschprung’s as a child is not overlooked, as this should prompt genetic testing. Although a family history may be helpful in identifying these patients, 5–9% of patients with HD possess de novo mutations, so you cannot rely on a negative family history [4]. If genetic testing is positive, screening for MEN2-related tumors should be performed as specified in the appropriate guidelines [4, 5].

Our case demonstrates the importance of maintaining a high clinical suspicion for the MEN2A variant in those with Hirschprung’s. The identification of an adrenal nodule or thyroid nodule in a patient with known HD should raise red flags. Any adrenal incidentaloma in a Hirschprung’s patient should prompt workup for pheochromocytoma, particularly those with unenhanced density of >10 Hounsfield units [6, 7]. Additional imaging characteristics may include avid enhancement with contrast, delayed washout, calcifications, and central hemorrhage or necrosis [7]. Although many patients would undergo biochemical screening for pheochromocytoma in the setting of an adrenal nodule, screening with serum calcitonin is not the current standard for patients with thyroid nodules, and as a result would delay diagnosis. A history of long-segment or total colonic aganglionosis should also raise suspicion as this occurs at a higher proportion than the general population in patients with specific RET mutations [2]. In our patient, the diagnosis of an underlying genetic condition was not discovered until she presented for adrenal incidentaloma. Only with this presentation was her history of HD brought to light and the connection made to the possibility of MEN2A and associated endocrine tumors. This is an unusual presentation as the diagnosis of a pheochromocytoma typically occurs after MTC in MEN2A cases and is only the presenting diagnosis in 27% of cases [4, 8].

Had our patient received genetic testing as a child, we likely would have prevented her development of medullary thyroid cancer as well as in her mother and possibly in her mother’s siblings. Her mother’s bilateral pheochromocytomas may have been discovered at a smaller size at a time when she could have been a candidate for a cortical sparing adrenalectomy.

Patients with Hirschprung’s have a higher risk of possessing a germline RET mutation and MEN2A. The risk is the highest in those with familial Hirschprung’s, as well as those with long-segment Hirschprung’s or total colonic aganglionosis. Incidental finding of an adrenal lesion in a patient with known Hirschprung’s should warrant careful consideration for pheochromocytoma. Genetic testing for germline RET mutations can help identify these patients and allow for appropriate screening for endocrine tumors, particularly medullary thyroid cancer.
